# Can the ETView VivaSight SL Rival Conventional Intubation Using the Macintosh Laryngoscope During Adult Resuscitation by Novice Physicians? A Randomized Crossover Manikin Study: Erratum

**DOI:** 10.1097/01.md.0000467313.46137.65

**Published:** 2015-06-26

In the article “Can the ETView VivaSight SL Rival Conventional Intubation Using the Macintosh Laryngoscope During Adult Resuscitation by Novice Physicians? A Randomized Crossover Manikin Study”,^1^ which appeared in Volume 94, Issue 21 of *Medicine*, the order of the author names was incorrectly presented. Dr. Kurowski's name should have appeared first in the byline. The article has been corrected online.
